# Anaesthetic management of cerebral arteriovenous malformation hemorrhage during pregnancy: A case series

**DOI:** 10.1097/MD.0000000000032753

**Published:** 2023-02-03

**Authors:** Yong Ji, Yi Liang, Bin Liu, Yaxin Wang, Ling Li, Yan Liu, Yifan Feng, Nuo Dong, Wei Xiong, Hongli Yue, Xu Jin

**Affiliations:** a Department of Anaesthesiology, Beijing Tiantan Hospital, Capital Medical University, Beijing, China.

**Keywords:** case report, cerebral arteriovenous malformation, cerebral hemorrhage, neurosurgical anesthesia, pregnancy

## Abstract

There is no clear consensus guidance for anesthesiologists on how to manage patients with cerebral arteriovenous malformation (cAVM) rupture and hemorrhage during pregnancy who need craniotomy. Our objective was to review the anesthesia management of pregnant women who underwent resection of cAVM at our institution and to provide opinions and suggestions.

**Patient concerns:**

Herein, we report of 3 patients with cAVM rupture and hemorrhage during pregnancy who underwent neurosurgery at the 22nd, 28th, and 20th weeks of pregnancy.

**Diagnoses:**

All 3 patients were admitted to the emergency department of our hospital due to sudden symptoms. Subsequently, their head imaging results confirmed the rupture and hemorrhage of cAVM. The rupture and hemorrhage of cAVM during pregnancy has a low incidence and high mortality, which seriously endangers the safety of the mother and fetus. For this emergency condition, craniotomy for removing intracranial lesions and clear hematoma can result in a chance of a successful delivery. Especially in the second and third trimesters of pregnancy, the management goal of anesthesia is to ensure the maternofetal safety and to maintain continuous pregnancy.

**Interventions:**

This article describes the process of intraoperative anesthesia management and maternal-fetal outcomes and discusses the key issues for the anesthesia management of cAVM rupture during pregnancy, including considerations of physiological changes during pregnancy and anesthesia medication, intraoperative monitoring, the maintenance of hemodynamic stability, and the control of intracranial pressure, among other considerations. Resection of intracranial lesions should be performed whenever possible while maintaining the pregnancy for better maternal and infant outcomes.

**Outcomes:**

The operations of the 3 pregnant women were successfully completed under our detailed anesthesia planning and careful anesthesia management. All the patients recovered well after the operation, and underwent cesarean section to give birth smoothly.

**Lessons:**

The preservation of pregnancy under cAVM resection is a complex challenge for anesthesiologists, and these 3 cases provide an extensive amount of experience for anesthesia management in similar situations. Detailed anesthesia planning and careful anesthesia management by anesthesiologists are important guarantees for good maternal and fetal outcomes.

## 1. Introduction

Cerebral arteriovenous malformation (cAVM) is a congenital disease in which a tangle of dysplastic vessels is supplied by arteries and drained by veins without capillaries, thus resulting in the formation of a high-flow, low-resistance shunt between the arterial and venous systems.^[1,2]^ This pathophysiology results in an annual bleeding rate of 2.2% for unruptured arteriovenous malformations (AVMs) and an annual bleeding rate of 4.5% for ruptured lesions.^[3]^ Maternal physiological changes during pregnancy, such as increased cardiac output and elevated estrogen levels, can affect the structures of these blood vessels, thus making them more prone to rupture.^[4–6]^ Although cAVM rupture and hemorrhage during pregnancy are rare (0.6–3.5%),^[7]^ once these events occur, they can be fatal to pregnant women and fetuses. Studies have shown that severe AVM and aneurysm complications during pregnancy account for 5% to 12% of all maternal deaths and 17% of fetal mortality.^[8,9]^

For such patients, preoperative multidisciplinary consultation is very important. Multidisciplinary consultation needs to be conducted in a timely manner, which is jointly discussed by neurosurgeons, obstetricians and anesthesiologists, to determine the individual treatment plans and to ensure the safety of the mother and fetuses.^[10]^ In 1 study of 27 patients who developed intracranial hemorrhage during pregnancy without an immediate treatment of AVM, the rebleeding rate was 26%, which was much higher than the rate of 6% for nonpregnant patients in the first year after the first hemorrhage. Given the high bleeding risk associated with AVM, various treatment options must be considered. Currently, the main treatment options involving aggressive intervention include microsurgery, radiosurgery and endovascular embolization techniques.^[11]^

To treat cAVM while maintaining pregnancy as the goal, it is necessary to avoid exposure to radioactive materials and contrast materials as much as possible; therefore, microsurgery is the first choice of treatment. If the craniotomy is determined, the next issue is to determine the timing of the operation. The timing of surgery should be based on a balance between maternal and fetal risks, gestational age and the urgency of surgery,^[12]^ with options including neurosurgery being performed first and continuing the pregnancy (early or middle stages of pregnancy) after the operation until the fetus is mature; performing a cesarean delivery before the neurosurgery and performing the neurosurgery at 1 to 2 weeks after obstetric operation when the general condition is stable; and performing a cesarean delivery combined with emergency neurosurgery. Although there are no clear guidelines, 32 weeks is typically used as the deadline for safe delivery of the fetus.^[13]^ It is currently recommended that neurosurgery in pregnant women should be performed in the second trimester because of the lower risks of fetal loss and teratogenicity.

Herein, we describe 3 cases of cAVM rupture and hemorrhage in mid-pregnancy in our hospital, 2 of which were in a coma, which seriously threatened the lives of the patients. Therefore, surgical intervention for cAVM resection is required after comprehensive consideration. The goal of anesthesia management is to achieve the purpose of the maintenance of the pregnancy while successfully completing the surgical resection of the intracranial lesions.

## 2. Case presentation

### 2.1. Case 1

**Figure 1.**
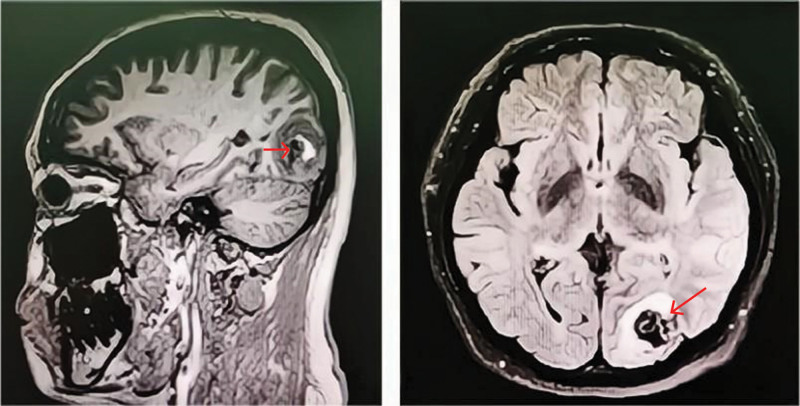
MRI of patient’s brain shows the lesion of AVM (arrow). AVM = arteriovenous malformation.

A 33-years-old woman (175 cm, 87 kg) at 22 weeks 5 days of gestation with twins presented to the emergency department with headache, nausea, vomiting and visual field defects. Magnetic resonance imaging of her brain revealed left occipital AVM with hemorrhage (Fig. 1). Her previous medical history included type 2 diabetes, which was treated via a subcutaneous injection of insulin. Multidisciplinary consultation consisting of neurosurgeons, obstetricians and anesthesiologists was organized to formulate a detailed treatment plan. When considering the possibility of the rebleeding of cAVM, which would be fatal to the mother and fetus, it was decided that a surgical resection of the intracranial lesion would be performed.

When the patient arrived in the operating room, she was connected to routine monitoring. Omeprazole (40 mg) was intravenously administered to the guttae, and dexamethasone (5 mg) was intravenously injected before the operation. Under local infiltration anesthesia with 2% lidocaine, arterial puncture was performed for invasive blood pressure (IBP) and arterial blood gas (ABG) analyses. The patient was preoxygenated with 100% oxygen (6 L/min) for 3 minutes before anesthesia. General anesthesia (GA) was induced with fentanyl (200 μg), propofol (100 mg), and rocuronium (50 mg) to complete tracheal intubation (size: 7.0). A right internal jugular vein puncture was performed before the patient was repositioned to the right lying position. A left auriculotemporal nerve block and bilateral greater and lesser occipital nerve blocks were performed with 0.5% ropivacaine to relieve pain during the surgical incision. Anaesthesia was maintained with 55% oxygen (2 L/min) and sevoflurane (1.5%). Remifentanil (0.05–0.1 μg/kg/min) was continuously infused during the operation, rocuronium was added intermittently to maintain the neuromuscular block and fentanyl was added as needed. In addition to routine intraoperative monitoring, fetal heart rate (FHR) monitoring was performed. Moreover, end-tidal carbon dioxide partial pressure was maintained at 30 to 35 mm Hg, and the bispectral index ranged from 40 to 60. The intraoperative FHR was maintained at 95 to 120 bpm without obvious signs of fetal hypoxia. The lesion was successfully resected, and the operation duration and anesthesia duration were 230 and 295 minutes, respectively. During the operation, the total blood loss was 300 mL, the urine volume was 600 mL, the total volume of sodium lactate Ringer’s injection was 2100 mL and the volume of 6% hydroxyethyl starch (HES, 130/0.4) was 500 mL. After the operation, the patient was extubated until she was fully awake. Subsequently, the patient was transferred to the intensive care unit (ICU) for rehabilitation treatment.

At the first day after surgery, the patient complained of incision pain. A patient-controlled analgesia pump was applied for pain relief, with 100 mL of normal saline containing 0.8 mg of fentanyl and 16 mg of ondansetron (background infusion: 2 mL/h; bolus dose: 0.5 mL/time; lockout time: 15 minutes). The patient-controlled analgesia pump was withdrawn on the third postoperative day because the visual analogue scale was less than 3 points. On the 6th day after the operation, the patient recovered well and was discharged without complications.

At 30 weeks of gestation, the patient gave birth to a pair of twins in a local hospital due to the premature rupture of membranes.

### 2.2. Case 2

**Figure 2.**
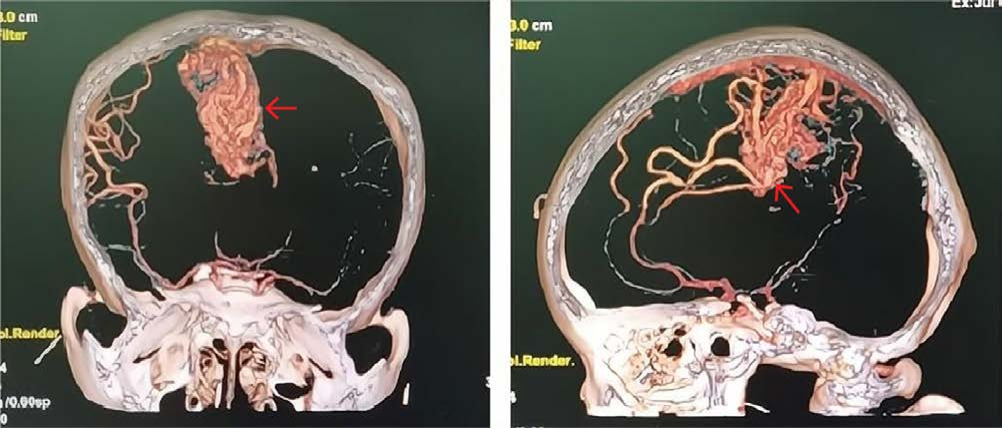
CTA of patient’s brain shows the lesion of AVM (arrow). AVM = arteriovenous malformation.

A 26-year-old woman (165 cm, 65 kg) presented to our hospital in the emergency department at 28 weeks 4 days of pregnancy due to headache, nausea, vomiting, blurred vision, slurred speech, limb weakness and limb convulsions. Computed tomography angiography of her brain revealed a cAVM with hemorrhage (Fig. 2). After admission, the patient underwent emergency intracerebral hematoma evacuation and cAVM resection.

The patient entered the operating room in a comatose state and had previously completed endotracheal intubation with a blood pressure (BP) of 119/63 mm Hg, a heart rate of 103 bpm and a SpO_2_ of 97%. GA was induced with sufentanil (10 µg), cis-atracurium (20 mg), etomidate (10 mg), and midazolam (5 mg). In addition, anesthesia was maintained with 80% oxygen (1.5 L/min) and sevoflurane (1–1.5%), as well as the intraoperative continuous pumping of cis-atracurium (6 mg/h) to maintain muscle relaxation and the intermittent addition of sufentanil (as needed) to maintain analgesia. In addition, patients underwent arterial cannulation to monitor IBP and internal jugular vein cannulation. To reduce painful irritation, local anesthesia was performed with 0.5% ropivacaine at the surgical incision site. Moreover, phenylephrine (0.1 µg/kg/min) was pumped after the operation to maintain the mean arterial pressure (MAP) at approximately 90 mm Hg. During the excision of the lesion, the amount of bleeding increased over a short time period, which resulted in the MAP decreasing to 62 mm Hg. The dose of phenylephrine was immediately adjusted, and fluid replacement therapy was performed. Intraoperative autologous blood recovery infusion was initiated, and allogeneic red blood cells and plasma were infused to maintain blood volume. After active treatment, the bleeding was well controlled, and the MAP gradually recovered to >80 mm Hg. Intraoperative continuous monitoring of FHR was maintained at 95 to 150 bpm. Furthermore, the operation lasted approximately 300 minutes. The blood loss during the operation was 1500 mL, the urine volume was 800 mL, the recovered autologous blood was 750 mL, the allogeneic red blood cell volume was 260 mL, the allogeneic plasma volume was 200 mL and the infusion volume was 2500 mL (sodium lactate Ringer’s injection 1500 mL + 6% HES 130/0.4 1000 mL). After the operation, the patient returned to the ICU with an endotracheal tube after the recovery of spontaneous respiration.

After the operation, the patient’s systolic blood pressure exceeded 140 mm Hg many times, the urine protein test changed from negative to “+ +” and the 24-hour urine protein quantification was 1098 mg. The preliminary diagnosis was severe preeclampsia. Therefore, the patient underwent cesarean section under GA 18 days after the operation (30+ weeks’ gestational age) and gave birth to a female infant (Apgar: 10 points), after which the mother and baby were discharged after 25 days of hospitalization.

### 2.3. Case 3

**Figure 3.**
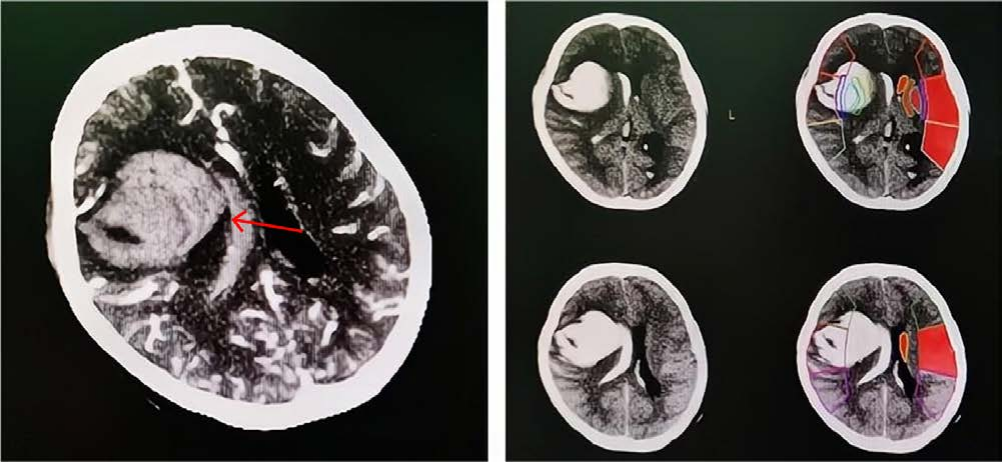
CT of patient’s brain shows the lesion of AVM (arrow). AVM = arteriovenous malformation.

A 29-year-old woman (155 cm, 55 kg) who was 20 weeks 6 days pregnant arrived at our hospital for emergency treatment due to sudden syncope. In combination with imaging examinations, right cerebral hemorrhage (AVM) combined with cerebral hernia was preliminarily diagnosed (Fig. 3). After admission, she underwent emergency craniotomy for removal and exploration of intracerebral hematoma and decompressive craniectomy.

The patient had slipped into a coma when entering the operating room and had completed endotracheal intubation before the coma event. The monitoring evaluation showed that the BP was 118/70 mm Hg, the heart rate was 91 bpm and the SpO_2_ was 98%. The premedication before anesthesia included midazolam (5 mg) and penehyclidine (1 mg). Sufentanil (20 µg), cis-atracurium (12 mg), and propofol (100 mg) were selected for anesthesia induction. In addition, anesthesia was maintained with 70% oxygen (2 L/min), as well as via propofol (6 mg/kg/h) by continuous infusion and remifentanil (0.15 µg/kg/min) to maintain analgesia. In addition, the patient underwent arterial cannulation to monitor IBP and ABG, and internal jugular vein cannulation was performed. The patient’s circulation was stable throughout the procedure. The duration of the operation lasted approximately 180 minutes. Furthermore, the blood loss during the operation was 300 mL, the urine output was 500 mL and the infusion volume was 3000 mL (sodium lactate Ringer’s injection 2500 mL + 6% HES 130/0.4 500 mL). When considering that the patient was complicated with cerebral hernia, 250 mL mannitol (50 g) was intraoperatively administered to reduce intracranial pressure (ICP), and there were no obvious immediate maternal or fetal complications. Postoperatively, the patient was returned to the ICU with a tracheal tube that was retained after her spontaneous breathing had resumed.

The patient was successfully discharged after 26 days in the hospital. At 37 weeks of pregnancy, the patient underwent cesarean section under GA and gave birth to a baby boy (Apgar: 10 points).

## 3. Discussion

In this article, we report of the anesthetic management of 3 patients with intracerebral hemorrhage induced by cAVM who underwent neurosurgery to remove the intracranial malformed vascular mass. There are few evidence-based recommendations for the management of neuroanesthetics during pregnancy. Therefore, it is necessary to rely on the general principles of obstetric and neurosurgical anesthesia to develop a successful anesthesia plan. Our main concerns include maternal and fetal safety and the avoidance of premature birth and abortion; therefore, the following challenges should be closely monitored.^[14]^

### 3.1. Preoperative considerations

For such patients, we should first initiate multidisciplinary consultation and formulate a detailed treatment plan. If surgery is needed, an adequate preoperative anesthesia evaluation should be performed, and relevant examinations should be improved. In addition, we should fully communicate with the patients and their families before the surgery, provide adequate psychological comfort and inform the individuals in detail about the risks of congenital malformations, fetal loss and premature birth.

### 3.2. Anaesthesia management

#### 1.3.2. Physiological changes during pregnancy

In the perioperative period, the following areas should be given special attention regarding the impact of physiological changes during pregnancy on anesthesia management. Airway: the airway mucosa of pregnant women is prone to edema. In addition, weight gain and breast enlargement can lead to laryngeal exposure and difficulties with endotracheal intubation. It is recommended to select a smaller endotracheal tube than the conventional tube for the operation. Respiratory system: due to the increase in oxygen consumption and the decrease in pulmonary functional residual capacity, the risk of hypoxemia in pregnant women will also significantly increase.^[15]^ Therefore, adequate preoxygen inhalation is required.^[16]^ Cardiovascular system: during pregnancy, BP and vascular resistance will decrease, blood volume and cardiac output will increase and the self-regulation of uterine blood vessels is defective. Therefore, it is necessary to be vigilant against hypotension after the administration of anesthesia. Aortocaval compression after 20 weeks of gestation should also be considered, and it is recommended to ensure the left inclination (15°) of the uterus during the operation to reduce compression. Gastrointestinal system: after 16 to 20 weeks of pregnancy, when considering the relaxation of the lower esophageal sphincter, the anatomical degeneration of the stomach and pylorus and the increase in intragastric pressure caused by the pregnant uterus, pregnant women are more likely to have reflux aspiration.^[17]^ Therefore, gastric acid secretion inhibitors and 5-HT_3_ inhibitors are recommended before the induction of GA.^[18]^ Blood and coagulation: relative haemodilution and hypercoagulability during pregnancy can lead to delayed appearance of typical signs of low blood volume and the easy occurrence of deep venous thrombosis, which requires close attention and active prevention.^[19,20]^ Central nervous system: the sensitivity of pregnant women to opioids and inhalants is increased, and the lowest alveolar effective concentration of inhaled anesthetics decreases by approximately 30% after 8 to 12 weeks of pregnancy; therefore, the dose of anesthetics should be appropriately reduced.^[21]^

#### 2.3.2. Intraoperative monitoring

Intraoperative management is crucial and helpful. In addition to routine monitoring, an arterial catheter should be used to monitor IBP and to facilitate the detection of ABG. If possible, FHR and uterine tension are recommended throughout the operation.^[22,23]^ Foetal bradycardia less than 80 bpm for 2 minutes indicates fetal hypoxia damage, but it is necessary to analyze the cause and to take corresponding measures according to the specific situation.^[24]^ In addition, it is best to assist with an ultrasound examination to ensure the health of the fetus.

#### 3.3.2. Maintenance of stable haemodynamics

It is vital to maintain haemodynamic stability.^[25]^ The balancing of adequate cerebral perfusion pressure and adequate uteroplacental perfusion pressure is a significant challenge.^[14]^ Due to the lack of automatic regulation of the uteroplacental circulation, any decrease in maternal arterial pressure will damage the uteroplacental blood flow, thus leading to fetal ischemia. Hypotension and hypovolemia should be avoided due to the fact that cAVM resection is often accompanied by massive intraoperative blood loss, which can be achieved through appropriate fluid replacement, the rational application of vasoactive drugs and the application of autologous blood recovery technology. Compared with ephedrine, phenylephrine is considered to be the vasopressor of choice that is preferred for better maternal cardiovascular stability and improved neonatal acid-base status.^[26]^ As reported in Case 2, the patient had a large amount of bleeding. We promptly initiated autologous blood recovery and transfused allogeneic red blood cells and plasma, as well as adjusting the dose of phenylephrine, to maintain BP and to ensure placental perfusion and cerebral perfusion. In addition, when considering the phenomenon of aortocaval compression by the enlarged uterus during pregnancy, maternal positioning should effectively displace the gravid uterus to the left to ensure the amount of returned blood volume and to reduce the occurrence of hypotension.^[23]^

#### 4.3.2. Decreasing intracranial pressure

If the pregnant patient is complicated with elevated ICP, the current general measures for treatment, such as mannitol, dexamethasone and mild hyperventilation, are reasonable.^[27,28]^ Mannitol is widely used in neurosurgery, but the use of mannitol in pregnant women will slowly accumulate in the fetus, and fetal hypertension will lead to physiological changes, such as decreased production of fetal lung fluid, decreased renal blood flow and increased blood sodium concentration.^[26]^ In individual case reports, mannitol administration at a dose of 0.25 to 0.5 g/kg has been used and appears to be safe. According to the study by Pooya Kazemi, higher doses of mannitol (0.8–1.7 g/kg) were used during the perioperative period, and there was no significant harm to the fetus.^[27]^ Herein, we reported that mannitol (0.9 g/kg) was used in Case 3, and no adverse events occurred. Therefore, mannitol can also be used to reduce ICP when necessary. Moreover, dexamethasone has been shown to improve useful neurological function and to prolong life in patients with intracranial hypertension caused by brain tumors; additionally, it has been proven to be effective in preventing postoperative nausea and vomiting.^[29]^ In addition, studies have shown that dexamethasone can significantly reduce rebleeding after subarachnoid hemorrhage.^[30]^ Dexamethasone is widely used in obstetrics because it can promote fetal lung maturation and reduce the incidence of neonatal mortality, respiratory distress syndrome, intraventricular hemorrhage and early neonatal infection.^[31]^ In conclusion, the use of dexamethasone is beneficial. It should be noted that if dexamethasone is used in the perioperative period, there is a risk of hyperglycemia. Therefore, the blood glucose concentration must be closely monitored. Appropriate hyperventilation after GA intubation can reduce ICP, but hyperventilation (<25 mm Hg) may cause uterine artery constriction and can shift the maternal hemoglobin oxygenation curve to the left.^[32]^ Therefore, it is recommended to maintain PaCO_2_ at 25 to 30 mm Hg for the mother.^[32]^

#### 5.3.2. Avoiding premature birth and abortion

We aim to maintain the pregnancy state while performing an uncomplicated neurosurgery procedure. Therefore, in terms of anesthesia management, we should ensure the safety of the mother and the fetus, reduce stress reactions and avoid premature birth and abortion. It is also necessary to ensure uterine relaxation to avoid uterine contraction caused by pain, surgical position compression and other stimulations of the uterus, which can lead to premature birth or abortion. Therefore, adequate analgesia should be ensured. For neurosurgery, preoperative and postoperative scalp nerve blocks can also be attempted.^[25]^ When preterm birth occurs, anti-uterine contraction agents can maintain pregnancy, but the preventive application is still controversial. Volatile anesthetics can inhibit uterine contraction, which may help to relax the uterus and prevent preterm birth.^[17]^ Anti-uterine contraction drugs include (but are not limited to) cyclooxygenase inhibitors, β-receptor agonists, calcium channel blockers and magnesium sulfate, among other drugs.^[33]^

#### 6.3.2. Consideration of anesthetic drugs

The use of anesthetics should be carefully considered. Although there is no clear evidence of teratogenic effects of anesthetics, pregnant women should theoretically use anesthetics as little as possible and use the lowest clinically effective concentration.^[33–35]^ Intravenous anesthetics (as represented by propofol) can quickly pass through the placenta; however, due to their rapid metabolism, the short-term use of these drugs has little impact on pregnant women and fetuses. Moreover, sevoflurane and desflurane are relatively safe in clinical dosages, but high concentrations may cause adverse hypotension. In addition, opioids can quickly pass through the placenta; therefore, drugs with short action times and rapid metabolism should be selected as much as possible. Moreover, muscle relaxants have little effect on the fetus because they have difficulty in passing through the placenta.^[36]^ Among our 3 reported cases, drugs such as fentanyl, sufentanil, remifentanil, propofol, midazolam, sevoflurane and other drugs were used without adverse effects.

#### 7.3.2. Anaesthesia recovery

Recovery after anesthesia requires close monitoring (especially concerning the airway and respiratory system) because the most serious anesthesia complications caused by insufficient ventilation or airway obstruction occur during induction, extubation or recovery.

### 3.3. Postoperative care

Insufficient postoperative analgesia will also increase the risk of preterm birth; however, the choice of analgesic drugs during pregnancy should be made with caution to avoid affecting the mother and fetus.^[21]^ During pregnancy, it is recommended that low-dose, weakly addictive opioids be used over a short period of time. After 32 weeks of pregnancy, the long-term use of nonsteroidal anti-inflammatory drugs should be avoided because such drugs may lead to the premature closure of the fetal ductus arteriosus.^[37]^ In addition, long-term exposure to nonsteroidal anti-inflammatory drugs may also cause damage to the fetal kidney, lung and cardiovascular systems. Local nerve blocks can also provide good postoperative analgesia and reduce the use of opioids; however, attention should be given to the control of the toxic doses of local anesthetics.

In our case report, gravidas with AVM rupture and hemorrhage should be comprehensively assessed before surgery. Pregnant patients and fetuses can benefit from a multidisciplinary consultation to develop a detailed plan. It is hoped that our case report can provide a reference for neuroanesthesia during pregnancy.

## Author contributions

**Conceptualization:** Yong Ji, Xu jin.

**Data curation:** Nuo Dong.

**Investigation:** Yan Liu, Yifan Feng, Wei Xiong.

**Writing – original draft:** Yi Liang, Bin Liu.

**Writing – review & editing:** Yaxin Wang, Ling Li, Hongli Yue.
